# Changes in the policy environment for infant and young child feeding in Vietnam, Bangladesh, and Ethiopia, and the role of targeted advocacy

**DOI:** 10.1186/s12889-017-4343-3

**Published:** 2017-06-13

**Authors:** Jody Harris, Edward A. Frongillo, Phuong H. Nguyen, Sunny S. Kim, Purnima Menon

**Affiliations:** 1 International Food Policy Research Institute, Washington, DC USA; 2 University of South Carolina, Columbia, SC USA

**Keywords:** Policy, Advocacy, Infant and young child feeding, Child undernutrition, Bangladesh, Vietnam, Ethiopia

## Abstract

**Background:**

There is limited literature examining shifts in policy environments for nutrition and infant and young child feeding (IYCF) over time, and on the potential contribution of targeted advocacy to improved policy environments in low- and middle-income countries. This study tracked changes in the policy environment over a four-year period in three countries, and examined the role of targeted nutrition and IYCF advocacy strategies by a global initiative.

**Methods:**

Qualitative methods, including key informant interviews, social network mapping, document and literature review, and event tracking, were used to gather data on nutrition and IYCF policies and programs, actor networks, and perceptions and salience of nutrition as an issue in 2010 and 2014 in Bangladesh, Ethiopia, and Vietnam. Theoretical frameworks from the policy sciences were used to analyze policy change over time, and drivers of change, across countries.

**Results:**

The written policy environment improved to differing extents in each country. By 2014, the discourse in all three countries mirrored international priorities of stunting reduction and exclusive breastfeeding. Yet competing nutrition priorities such as acute malnutrition, food insecurity, and nutrition transitions remained in each context. Key actor groups in each country were government, civil society, development partners and the private sector. Infant formula companies, in particular, emerged as key players against enforcement of IYCF legislation. The role of a targeted IYCF advocacy and policy support initiative was well-recognized in supporting multiple facets of the policy environment in each country, ranging from alliances to legislation and implementation support. Despite progress, however, government commitment to funding, implementation, and enforcement is still emerging in each country, thus challenging the potential impact of new and improved policies.

**Conclusion:**

Targeted policy advocacy can catalyze change in national nutrition and IYCF policy environments, especially actor commitment, policy guidance, and legislation. Implementation constraints – financing, capacity and commitment of systems, and competing priorities and actors – are essential to address to sustain further progress. The lack of pressing political urgency for nutrition and IYCF, and the uncertain role of international networks in national policy spaces, has implications for the potential for change.

## Background

Child undernutrition and infant and young child feeding (IYCF) are complex issues requiring action from many different sectors of society [1]. Policymakers worldwide often do not recognize the impact of sub-optimal feeding practices on child undernutrition, survival, educational potential, and economic development, and instead view undernutrition solely as a food-security issue that results from poverty. These views lead to underinvestment in interventions to improve health and nutrition behaviors, and lack of implementation, monitoring, and funding of policies and associated regulations hinder the effectiveness of IYCF interventions that many see as crucial.

Creating an “enabling environment” for good nutrition has been defined as “political and policy processes that build and sustain momentum for the effective implementation of actions that reduce undernutrition” [2]. Preceding policy formulation and implementation, the formation of a coherent discourse to set policy agendas is central to policy-making processes [3]. The framing of undernutrition as an issue--ideas shaping the narrative or persuasive understanding of undernutrition, as well as understanding of the determinants or consequences of undernutrition--through advocacy can therefore change the course of a policy discussion [4]. Low issue salience, poor advocacy, and lack of definition of nutrition among different actors are issues for nutrition, however [5], and this purposive framing is often overlooked or done poorly [6], allowing differences in views among actors, particularly those from different sectors, to become an insurmountable barrier to change [7, 8]. Priority of an issue in most contexts is assigned by political leadership, and government and politics in the policy process is central [3, 5, 9, 10], but engagement of civil society can also change the course of a policy [10].

Aiming to address issues of priority, agenda-setting, and improved policy for IYCF and child undernutrition, Alive & Thrive (A&T) supported policy advocacy alongside large-scale social and behavior change communication interventions in three countries (Vietnam, Bangladesh, and Ethiopia) for a 6-year period (2009–2014). The intervention was an example of policy advocacy, defined as “an intervention intended to catalyze, stimulate or otherwise seed some form of change through different forms of persuasion” [11]. A&T’s overarching model was based on the assumption that sustainable improvements in breastfeeding and complementary feeding can be achieved through strategies to: 1) improve the policy and regulatory environment to support IYCF interventions and practices; and 2) create, shape, and support demand for improved IYCF social norms and practices at the community and family levels. To improve the IYCF policy and regulatory environments, A&T used targeted advocacy based on international evidence and best practice to raise policymaker and civil-society understanding of IYCF issues and to initiate dialogue, garner consensus and commitment, and build the capacity of national organizations to address gaps in the policy and regulatory environment.

This paper focuses on assessing the contribution of targeted advocacy in improving the enabling environment for IYCF and child nutrition in the three A&T countries. We do this not as a direct attribution-focused analysis, but by taking the broader perspective of documenting what changed in the policy landscape for nutrition and IYCF over the duration of A&Ts involvement, attempting to assess why the policy landscape changed, and to document potential contributions made by A&T to the changed policy environment.

## Methods

In-depth qualitative approaches are necessary for assessment of complex advocacy interventions, with challenges in doing so, including assessment of causal relationships, definition of success, long horizons, and changing contexts [11]. In this study, mixed qualitative methods, including document and literature review, social network mapping, key informant interviews, and event tracking, were used to gather data on the existence of policies and programs, policy actor networks, and perceptions and salience of nutrition as an issue at two time points in each country, as well as on A&T’s targeted advocacy activities. In this way we documented changes over several years in policy and legislation in support of child nutrition and IYCF, and examined the contribution of the intervention as recalled by respondents.

### Data sources

Data were from multi-method assessments conducted in 2010 and 2014. Specific methods are described below:

Document and literature review

National policy documents and institutional frameworks were reviewed in 2010, providing a broad narrative synthesis of current policy and legislation relevant to nutrition. The document review was updated in 2014. Various nationally-representative surveys which cover aspects of nutrition and IYCF, such as the Demographic and Health Surveys (DHS), Multiple Indicator Cluster Surveys (MICS), or other national nutrition surveys and surveillance were also reviewed to provide background on context and nutrition outcomes.

Social network mapping

In each country, the landscape of policy actors relevant to nutrition policy was captured in 2010 and 2014 using the NetMap method. Net-Map is a participatory interview technique that combines social network analysis [12], stakeholder mapping [13] and power mapping [14]. Net-Map helps to examine the relative influence, goals and perspectives of various stakeholders, and examines how these stakeholders interact with each other. The method started by listing all the actors involved in child nutrition and IYCF policy issues at the national level, then asked interview participants to determine how actors are linked (through advocacy, technical assistance, and financial flows- only the advocacy links are shown here, illustrated with the case of Vietnam) and examine how influential and supportive each actor is in IYCF policy issues. Net-Map interviews were used to collect data from two groups: national and government actors (domestic), and expatriate groups including NGOs (international). In Bangladesh and Ethiopia, these groups were interviewed together, and in Vietnam separately, depending on the local policy context; in Vietnam, two distinct sets of stakeholders emerged regarding language comprehension and comfort in the interview, so interviews were done for each set separately. The social network mappings were conducted with representatives of the functional agencies related to the provision of evidence, development, and implementation of the policy environment for nutrition and IYCF. In Vietnam, one was conducted with national actors (6 participants from different ministries and departments of government) and one with international actors (7 participants from Save the Children, Health Bridge, World Bank, UNICEF, WHO, Plan International, and World Vision). In Bangladesh, mappings were conducted with 9 participants from medical associations, government, NGOs, research institutions, donor agencies, media, and civil society. Participants for mapping in Ethiopia came from government (e.g., Food Science & Nutrition, Federal Ministry of Health, Ministry of Women, Children & Youth Association) and international agencies (Alive & Thrive, Concern Worldwide, International Food Policy Research Institute, Save the Children, and World Bank).

Key informant interviews

Interviews with stakeholders in the nutrition and IYCF policy process at baseline and endline provided a deeper understanding on the leading actors in policy networks, how issues of nutrition and IYCF are perceived and acted upon in each country, and changes over time. Semi-structured interview guides at the national level included questions on the current situation and major changes perceived in the nutrition and IYCF policy landscape; challenges to achieving an enabling environment for improved IYCF; drivers of change in nutrition and IYCF policy and implementation, and potential ways forward; and the perceived roles of different policy actors, including A&T. At the sub-national level, interview guides asked about the impact of national policy and guidelines on nutrition and IYCF action, and specific questions on the role of A&T. Interview respondents covered technical agencies, research organizations, civil society, government and other actors in each country, with only one respondent per agency or organization. The number of interviews was approximately 20–25 interviewees, at each point in time, in each country. We interviewed nutrition-related stakeholders such as implementers, development partners, government organizations, and media agencies. Interview participation was affected by scheduling challenges, but there were no recorded refusals; some interviewees did not want to be tape recorded. Information saturation and coverage of major interviewee categories were criteria for deciding on when to stop interviews.

A&T program document review and event tracking database

Detailed tracking of A&T activities and events in each country, extracted regularly from A&T’s routine monthly partner updates, was captured in an ongoing database file. A synthesis report was created from this database, covering key trends and themes in A&T activity. Activities in the database were coded according to advocacy best practices [15] to assess whether activities included such best practices as training, capacity building, alliance building, media engagement, policy communication, etc. The strategies used in each year were summarized and examined in the analysis for this paper.

### Analytic frameworks

Policy processes for multifaceted issues are complex, with many moving parts. A view of the policy process is provided through seeing the process as encompassing broad stages of agenda-setting, policy formulation and adoption, implementation, and review [16]. Within stages, different frameworks look in more detail at different parts of the policy process. Our study was informed by a commonly used framework for agenda-setting on factors that influence prioritization of international public health issues [17–19], and by ideas on commitment to policy creation and implementation for nutrition [20].

**Fig. 1:**
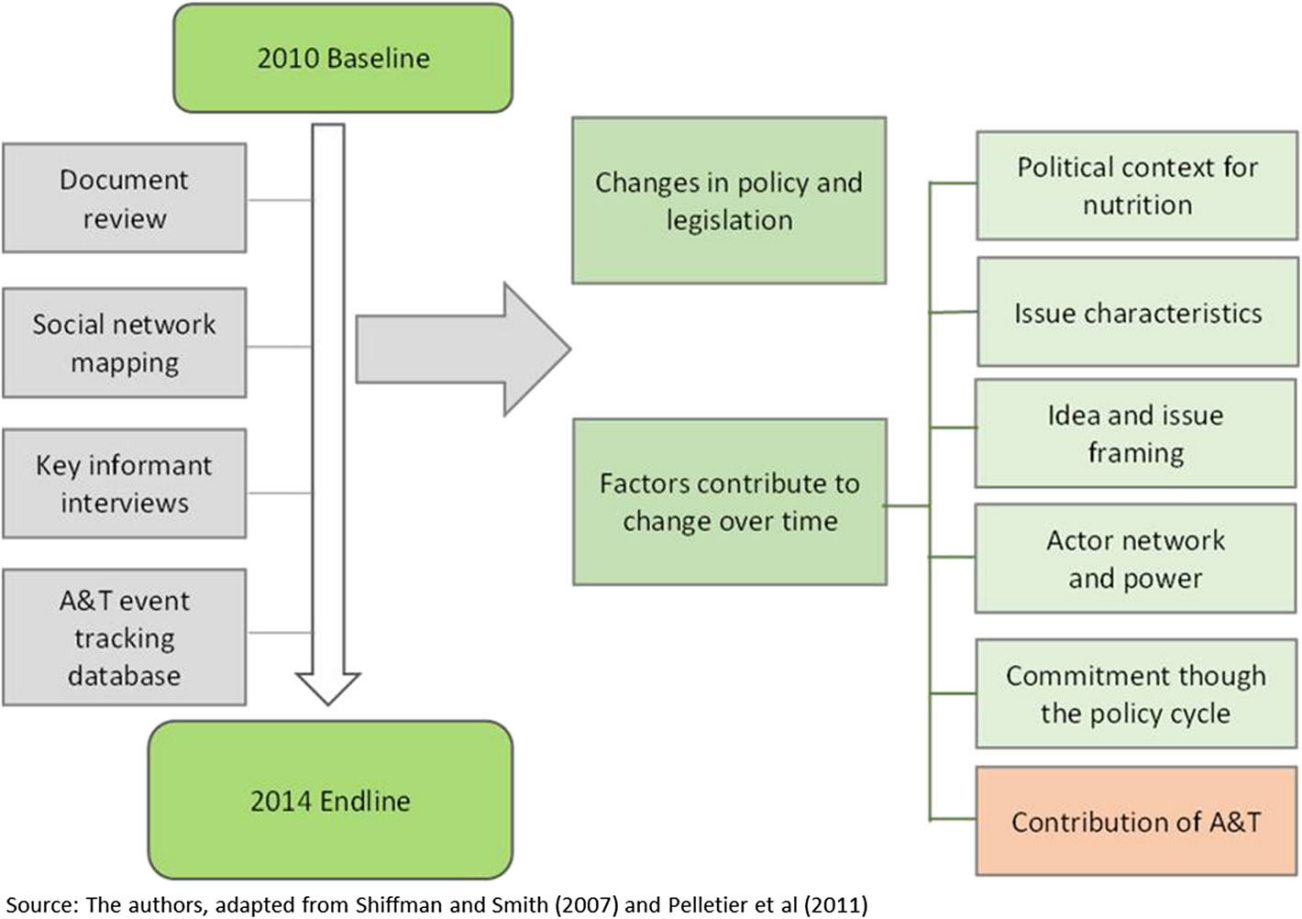
Conceptual framework

The Shiffman and Smith framework recognizes key factors that influence prioritization of issues, comprising actors and their power (including leadership and guiding institutions, and civil society mobilization); ideas and issue framing (including the internal frame within the policy community, and external frame more broadly); political context (including governance structures and focusing events); and the nature of the issue itself (including relevant outcomes and existing policy). Recent work has used adaptations of this framework successfully for assessing nutrition policy processes [8, 21]. Beyond agenda-setting, Pelletier, Frongillo and colleagues [8] have emphasized the role of commitment of systems set up to make and implement policy to move the nutrition policy agenda. We used, therefore, an adaptation of the framework developed by Heaver [20] to examine high-level attention, political commitment in terms of goals and policies, and system-wide commitment as defined by allocation of funds and authority. Each of these issues is incorporated in our overall analytic framework (Fig. 1).

### Data analysis

NVivo 10 software was used to organize, code, and analyze all interview data, and Vizualyzer software was used to create and analyze the NetMaps. The 2014 network analysis for Bangladesh used a different software package [22]. Initially working within each country, data from key informant interviews were coded to themes based on the guiding frameworks and accommodating emerging themes as they arose. Frequency of reference by respondents was used to identify prioritized issues, and contradictory perspectives were also highlighted where these existed. For the social network mapping, qualitative analysis of network content and structure was undertaken, to assess the network shape and links between actors, the roles and positions of key actors and shifts over time. Policy documents were read and summarized, looking particularly for elements affecting nutrition and IYCF. The events database was synthesized into core action areas and these were summarized in tables showing the types and frequencies of activities undertaken by A&T. Data from these four sources were combined into country-specific narrative synthesis reports, organized by the key elements of the analytic frameworks.

The country reports were then subjected to a form of framework analysis, a method designed for applied qualitative policy research [23]. In this analysis, findings were synthesized first within and then across countries in a formal and consistent manner, reducing the data down to core elements with reference to the analytic frameworks, to draw out similarities and differences among the countries. A configurational approach to understanding causation, where combinations of causes interact to contribute to an effect [11], was used to articulate tentative findings and conclusions on how policy changes happened.

## Results

### Changes in policy and legislation

Nutrition and IYCF policy environments have evolved differently in each country, though they are linked by the use of common evidence to inform policy, and the common goals of the international community working across countries.

Nutrition policies in Vietnam, even before 2010, have for the most part been logical in progression. Since 2010, they appear to have become even more coherent in directing complementary actions by different sectors and agencies. The year 2012 marks a key point for change in the national policy environment for IYCF, including the approval of two legal policies. One was an amendment to the Code of Labor that allows mothers to take six months paid maternity leave, improving legislation for mothers working in the formal sector. The other was an amendment to the Law on Advertising that bans advertisements for breast milk substitutes, aiming to address the persistent Code violations that have been seen as a key public health issue. The written policy environment for nutrition and IYCF in Vietnam is, thus, now one of the most promising in any high-burden country. Throughout the succession of policies and plans, however, there is a pattern of technical completeness but practical under-stipulation. Although the programs and activities prescribed are those which would improve IYCF and eventually reduce stunting if implemented, detailed implementation plans are rare and funding plans rarer still.

In Bangladesh, the political context and resulting policies became increasingly nutrition-focused over the four years of the study, with several major policy updates and changes. Most notable among these was the emergence of the National Nutrition Services (NNS) in 2011, aiming to mainstream nutrition services into the health system under the Health, Population and Nutrition Sector Development Programme. In addition, updates were made to the Regulation of Breast Milk Substitutes Marketing Act (2014), the National Communication Framework and Plan for Infant and Young Child Feeding in Bangladesh (2010–2013), and The National Food Safety and Quality Policy and Plan of Action (2012). These policies and laws were seen as significant strides forward, and the mainstreaming of policy led to the development of standardized tools utilized in programs. The promises of policy have not yet translated to action effectively. A recent study [24] highlights several challenges in the implementation of the NNS. Interviewees noted, with regard to the amendment to the Code, that formula companies remain powerful and violations rife.

In Ethiopia, a key policy change since 2010 was the revision of the National Nutrition Program (NNP), launched in 2014, with more attention given to reducing stunting as an outcome, and to multi-sectoral involvement and nutrition-sensitive interventions. This revision process started with the Accelerated Stunting Reduction Strategy in early 2010 that focused on maternal and adolescent nutrition, IYCF, and capacity building, issues neglected in the original NNP. The content of the revised NNP is considered technically sound, and the document incorporated many evidence-based recommendations from international actors, especially an emphasis on prevention of chronic malnutrition alongside measures to treat severe acute malnutrition. The execution of the strategy outlined in the NNP at local levels, where implementation takes place, is considered to face multiple challenges [25]. Similarly for IYCF, while there is a high-level strategy in place, it lacks completeness (for instance ratification of the Code), and a challenge has been a lack of updated IYCF guidelines for use by frontline practitioners.

### Factors contributing to change over time

The sections below concentrate on analyzing the five domains in our analytical frameworks to examine how these factors may have contributed to the changes seen over time. We focus on the agenda-setting domains of political contexts, issue characteristics, actor power, and issue framing, and on commitment throughout the policy cycle (Table 1).

**Table 1:** Overview of findings

	Vietnam	Bangladesh	Ethiopia
Changes in policy and legislation			
Policy changes	• Revision of the formula advertising law • Revision of the maternity leave policy • National Nutrition Strategy • IYCF Action Plan ◦ Technical completeness but practical under-stipulation	• Replacement of the NNP with the NNS • Revision of legislation on breastmilk substitutes ◦ Not succeeded in translation to action in all cases; challenges remain in rollout	• Revision of the NNP ◦ Strong content, but dissemination to local levels is not moving quickly ◦ Other key actions still lacking, such as BFHI and ratification of the Code
Agenda setting and commitment: Why did it change?			
Political context	• National Institute of Nutrition, under MOH, hosts the secretariat for the National Nutrition Strategy • Nutrition Cluster Group engages non-government actors • Vietnam’s nutrition programming is executed through a decentralization process	• National Nutrition Coordination Body is largely inactive; Nutrition Working Group and SUN-related bodies liaise with government • National IYCF alliance group was initiated during the time-frame of A&T	• National Nutrition Coordination Body sits within MOH • Nutrition Development Partner Group engages non-government actors
Issue characteristics	• Low breastfeeding rates with limited change over time offers advocacy opportunities • Improvements in stunting and complementary feeding make it difficult to position nutrition as a priority	• Breastfeeding, complementary feeding, and stunting are significant challenges presenting key advocacy opportunities	• Low rates of appropriate complementary feeding and high stunting levels, meant that advocacy focused primarily on these issues; breastfeeding rates are high
Ideas and issue framing	• Opinions on what should be addressed are not harmonized: Formula marketing and maternal employment and leave continue to be large issues • Stunting and IYCF are generally thought of as a chosen issue, not immediately pressing politically; the international community have chosen these issues • It is unclear how far new attention to stunting and IYCF extends beyond international actors and their immediate contacts in the national policy community, to the rest of government or to the population	• Integration of ideas on nutrition-specific and nutrition-sensitive initiatives is embedded • The importance of accounting for geographical differentiations within Bangladesh is highlighted • Natural disaster rates mean emergency nutrition continues to require attention • It is unclear how ideas related to IYCF are presented to actors outside of the nutrition policy community	• Pronounced change in the nutrition discourse within the policy community, to encompass stunting, though both stunting and wasting remain priority issues • Government priority setting has been slow to move in line with international stunting and IYCF agendas • It is unclear whether this broader understanding of nutrition is reaching to those working in nutrition beyond the policy community; on the ground, the focus remains on programs to address wasting and food insecurity
Actor networks and power	• Actor networks are more complex in 2014 • MOH continues to be regarded as influential, while NIN appears to be regarded as less central • UNICEF and formula companies are clear hubs in the international map, while government institutions remain central in the domestic map • Key players driving for pro-IYCF change remained the international community; There has not been strategic leadership from within national government	• Perceived increase not only in activity level but also in the number of actors • Government, individual leaders and media were reflected centrally; MoHFW is the most influential player • Formula companies are a powerful group of actors, with effective media campaigns as well as elite alliances, with little change since 2010 • Individual leadership remains lacking	• Little has changed over the past five years in terms of actors and their power in Ethiopia • FMOH is still the hub, because of their mandate, and government has convening power • Civil society and donors are numerous and active in Ethiopia, but are weak in influence due to restricting regulations
Government commitment	• High-level government attention has been paid, and written policy changed • Lack of clear implementation and enforcement plans • Lack of government-allocated funding	• Major shift towards government leadership on nutrition policy and implementation • Most financial support for nutrition policy still comes from international sources	• Government attention to nutrition is sporadic, and limited to certain sectors • The government funding situation for nutrition is poor; funding for nutrition is mainly through international donors
Role of Alive & Thrive			
	• A&T along with UNICEF was often cited as being among the most influential and active policy actors • In general, those involved in IYCF policy advocacy in Vietnam recognized A&T’s role as pivotal	• A&T is credited with successful media campaigns, which recognized and supported the central role of the government • Challenges include sustainability (particularly of funding) and integration of related issues into nutrition programming	• A&T’s contributions in BCC and media campaigns, IYCF messages and materials, and input to the NNP were widely recognized, contributing in the shift of policy and programs to a stunting reduction focus • The project was perceived as taking on too much and achieving too little in terms of actual implementation

### Political context for nutrition

All three countries were signatories at the time of the study to the Millennium Development Goals (MDGs). Only Ethiopia was part of the Scaling Up Nutrition (SUN) movement for the entire duration, since 2010, with Bangladesh joining in September 2012 and Vietnam joining in January 2014.

In Vietnam, the convening body for nutrition is the National Institute of Nutrition (NIN) in the Ministry of Health (MoH). It has the Secretariat specifically for the effective implementation of the National Nutrition Strategy (NNS). The Nutrition Cluster Group is the multi-stakeholder platform which engages with key representatives from across sectors and external to the government, including development partners such as UNICEF and the WHO. Vietnam’s nutrition programming is executed through a decentralization process, where provinces develop annual nutrition plans which are then funded through allocated funding at the national and provincial level. In previous work we have examined this process [26–28].

The Bangladesh National Nutrition Coordination Body (NNCB) is chaired by the Minister of Health and co-chaired by the Ministers of Agriculture and Education. It is largely inactive, although other local technical working groups for nutrition are highly active. These include the Nutrition Working Group, which has a liaison role with the government, and a variety of SUN-focused groups including a donor group, civil society groups and more. A national IYCF alliance group was initiated during the time-frame of A&T, with support from A&T but anchored by the government and local partners. Several major development partners–USAID, DFID, and the World Bank in particular–made financial and technical collaborations with the government and other implementers over this period, thus assuring widespread availability of resources. UNICEF provided financial and technical support to the government through the Maternal and Young Child Nutrition Security Initiative in Asia.

Ethiopia also has a Nutrition Development Partner Group (NDPG) which engages UN agencies, donors and civil society. Several development partners are active financial contributors to Ethiopia’s efforts to improve nutrition and the health system, especially USAID and the Bill & Melinda Gates Foundation and the World Bank.

Overall, in all three countries, there were national coordination and leadership entities available, as well as a facilitating nutrition policy context, in some part due to the country’s own commitment and engagement with the SUN. These are likely, over time, to have created an environment conducive to absorb and engage with technically coherent messages of the policy advocacy supported by A&T for IYCF and nutrition as well.

### Issue characteristics

**Fig. 2:**
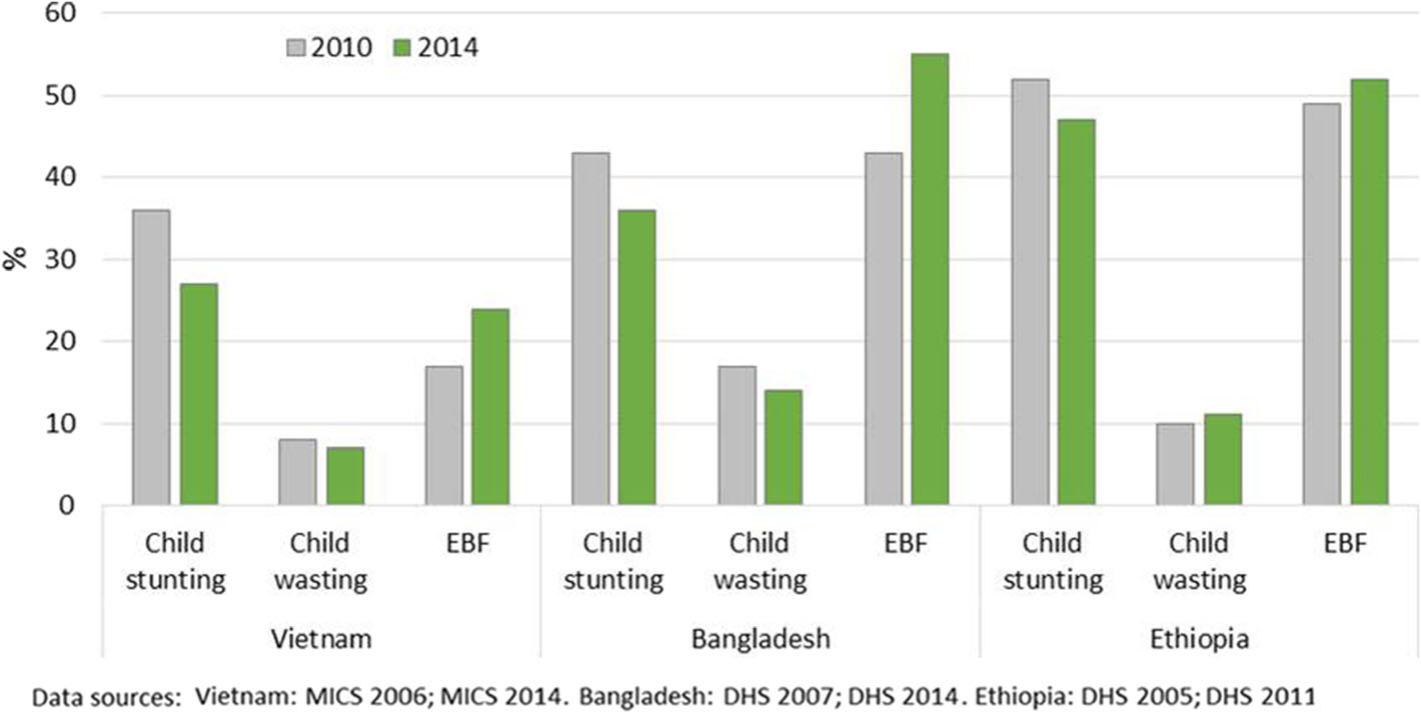
Nutrition and IYCF outcomes- Change over time in three countries

In all three countries, trends in key outcomes were broadly positive; over the six years of the A&T program, stunting reduced and exclusive breastfeeding increased in each context (Fig. 2). There were also differences between the countries which potentially affected the ability to advocate for attention to stunting as well as all IYCF practices. In Vietnam, breastfeeding offered huge opportunities for advocacy, given extremely low levels and limited change over time. Improvements in stunting and generally positive complementary feeding practices, however, made it somewhat more difficult to position these topics as political priorities. Nevertheless, in Vietnam, the advocacy community had been working on stunting reduction since about 2006 [21], thus paving the way for a further focus on IYCF, and especially the poor state of breastfeeding as a critical challenge. In Bangladesh, breastfeeding, complementary feeding, and stunting were significant challenges with high prevalence levels and all were addressed in the advocacy work. Despite changes over time, the lingering high levels of these outcomes ensure that advocacy groups can continue to call for attention to these over time. Ethiopia’s positive trends and high levels of exclusive breastfeeding, alongside abysmally low rates of appropriate complementary feeding and high stunting levels, meant that advocacy focused primarily on complementary feeding and stunting. Thus the characteristics of nutrition outcomes in each country made for different environments within which A&T aimed to bring increased attention to the issues of stunting and different aspects of IYCF.

### Ideas and issue framing

The positioning of stunting as a neglected aspect of undernutrition that needed attention was slow in Vietnam, starting to take shape before 2010 [21] but finding more momentum in discourse to 2014. While in 2010 nutrition was perceived as a health issue, interviews with the nutrition and IYCF policy community in 2014 revealed a high degree of consistency in terms of understanding the broader determinants of undernutrition, largely reflecting the nutrition narratives and evidence that had been emerging over time in the international nutrition community. Opinions on what actions should be addressed were not as harmonized as participants’ understanding of the causes and consequences of undernutrition. Marketing of infant formula and maternal leave continue to be large constraints in a modernizing workforce, and although policy is slowly shifting in favor of breastfeeding mothers, some actors continue to support use of formula in certain cases, perhaps empathetic towards the multiple demands placed on Vietnam’s working women. Whether this response comes from a real pragmatism, or is influenced by the formula company lobbying alluded to in several interviews, is not apparent from this work. Nevertheless, we identified a more nuanced understanding of the roles of breastfeeding and formula feeding in a modernizing society than is usually apparent in the discourse of the international public health community; this especially made for a continued lack of clarity and cohesiveness of goals between domestic and international actors. Shiffman and Smith [19] note the importance of local policy community cohesion in keeping an issue on the agenda and translating it into locally relevant action, but in this case local cohesion was not as clear-cut in Vietnam, for breastfeeding in particular.

In 2014, nutrition ideas and issue framing in Bangladesh were well-aligned with international standards and advocacy foci, including the MDGs and the SUN movement. In addition, the need to integrate nutrition-specific and nutrition-sensitive initiatives, seeing nutrition as a development issue, and the importance of accounting for geographical differentiations within the country, such as the urban–rural divide, in nutrition programming were highlighted in responses. Furthermore, as Bangladesh has frequent natural disasters, the implications of emergency situations for nutrition and IYCF were underscored. Exclusive breastfeeding and appropriate complementary feeding are the two topics stressed most by respondents working in the nutrition policy field in Bangladesh; exclusive breastfeeding in particular was considered to have had a history of conflict with the private sector, and interviewees perceived the need for the policy community to gain control over formula companies and their powerful marketing schemes that undermine IYCF best practices. Respondents expressed the knowledge that other economic interests are also at play in undermining IYCF best practices, such as labor laws calling for maternity leave that fail to be implemented on the ground. It is plausible that the engagement with the SUN movement, the coherence of the major development partners in framing nutrition narratives, and a globally engaged local research and technical community together contributed to the strong technically grounded narrative.

There has been a pronounced change over time in the nutrition discourse within the Ethiopian policy community to encompass stunting, in general, and to some extent the companion narratives on IYCF and the 1000 days. This is a broader understanding of nutrition, beyond the emergency and food aid narratives prevalent since the 1980s. Both stunting and wasting remain priority issues within the nutrition community, however, and actors particularly at lower levels struggle against strong prevailing discourses and ideas that have not moved quickly to align with international evidence and priorities. There is also a lack of clarity on the most effective interventions to address all of these issues simultaneously, which could limit advocacy. Respondents were not clear that understanding of the broader definition of nutrition action in the policy community had filtered through coherently to those working in nutrition beyond Addis Ababa, even though overall progress is noted on efforts to solidify multisectoral nutrition processes in Ethiopia [29]. On the ground, it was noted that the focus remains on programs to address wasting and food insecurity due to lack of time or resources for the new understanding to be disseminated, entrenched programs taking time to change course, and incomplete understanding on the part of both policymakers and implementers as to what exactly stunting reduction through multisectoral processes entails in practice. The importance of IYCF as a key input, however, to preventing stunting was well-understood to have evolved over this duration.

### Actor networks and power

**Fig. 3:**
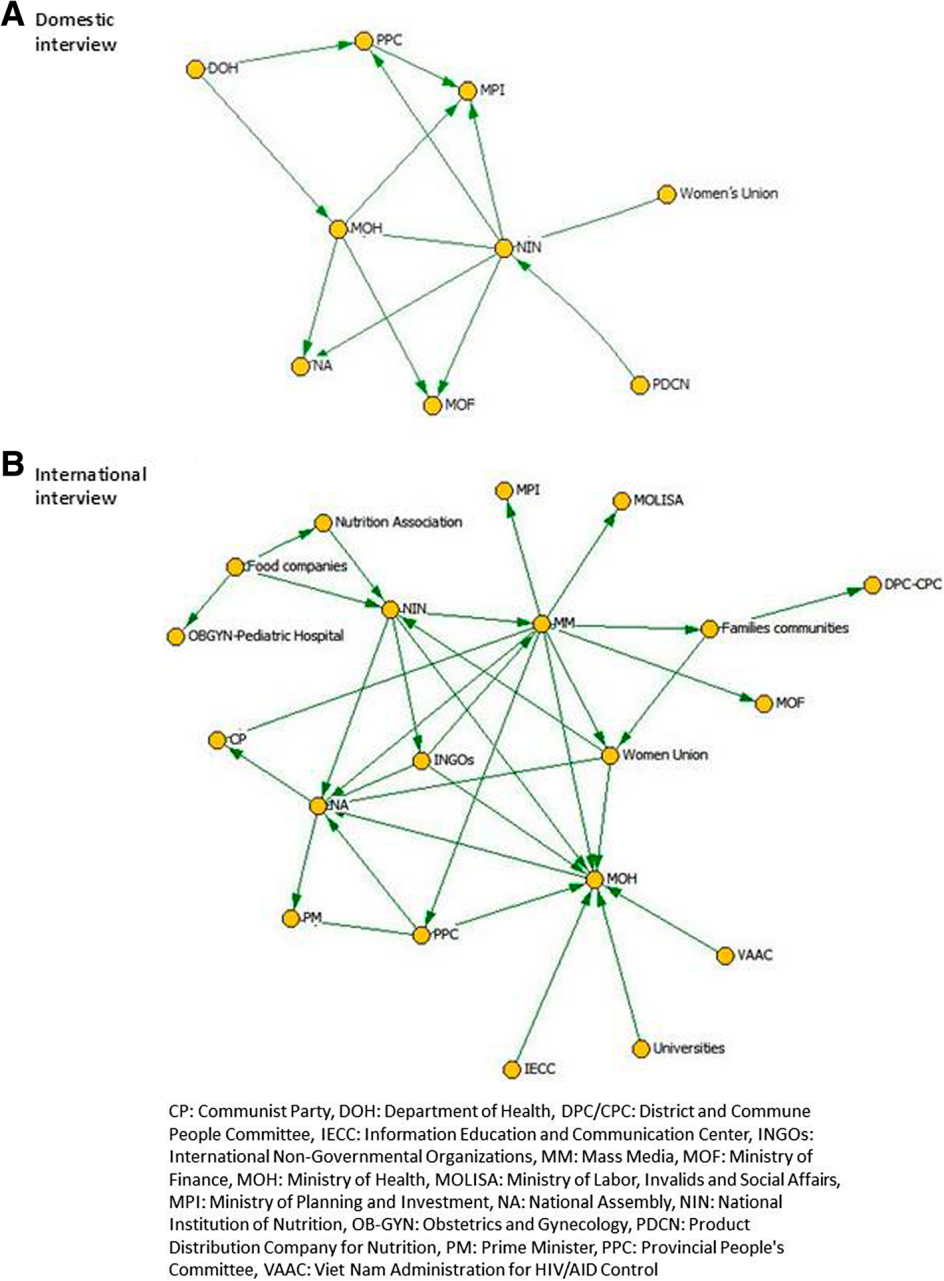
IYCF and nutrition actor network maps for Vietnam in 2010. **a** Domestic interview. **b** International interview

**Fig. 4:**
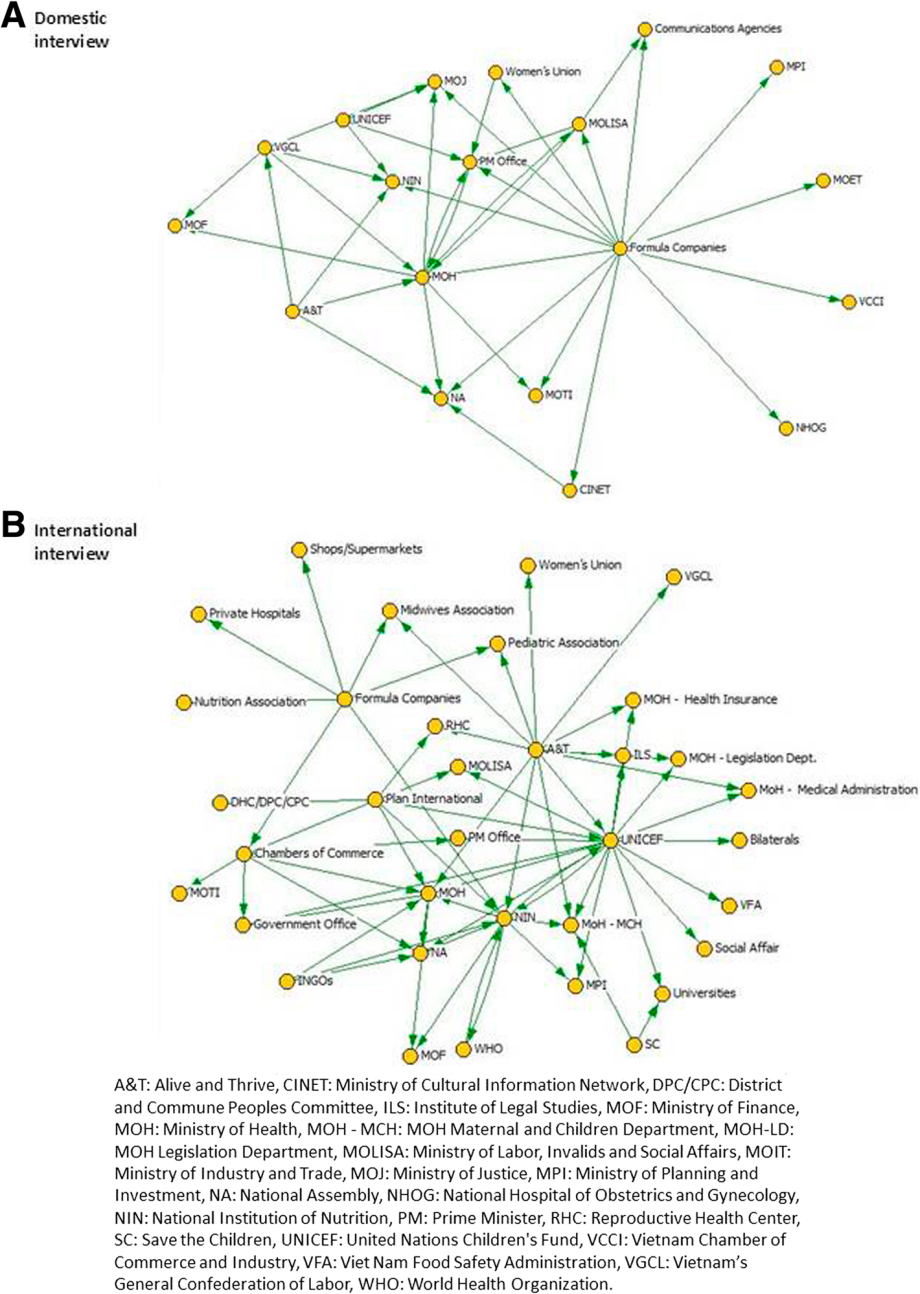
IYCF and nutrition actor network maps for Vietnam in 2014. **a** Domestic interview. **b** International interview

In Vietnam, in 2014 the actor networks were more complex than they were in 2010 (Figs. 3 and 4). In both domestic and international respondent maps the international organizations were specified individually in 2014, suggesting that their roles had become clearer and/or more differentiated to each other since 2010. Maps remained different between domestic and international respondents, however, with UNICEF and formula companies being clearer hubs in the international map, while government policy institutions remained central in the domestic map. This could indicate the different perspectives of the different groups, where international actors focus on changing discourse and norms, while domestic actors rely on formal policy change and related formal structures, or their differing priorities in terms of change. While formula marketing was understood to be an issue in 2010, formula companies were not mentioned on stakeholder maps then, yet they were central in 2014. Key players driving for pro-IYCF change remained the international community, but additional strong actors entered the scene, in particular A&T that, although unknown in 2010, was noted to have made a strategic alliance with UNICEF. A key finding is that there has been little strategic leadership from key guiding institutions within national government or from individuals. The Ministry of Health (MOH), the main nutrition policy body, continues to be regarded as influential, although the two networks disagreed on the supportiveness of MOH towards IYCF. International actors noted that the MOH often required external funding before acting on nutrition, and domestic respondents saw the MoH as a key player. The National Institute of Nutrition appears to be regarded as less central, powerful, or supportive in 2014, with their aims and incentives unclear to many respondents.

The shift from nutrition agenda led by an NGO to one led by the government nutrition is the most important change in Bangladesh during this study period (see NetMaps in Rasheed et al., this issue). While the combination of government, NGO, and donor efforts on nutrition reveal a multiplicity of actors working on this issue in Bangladesh, actors were generally perceived to cooperate on nutrition and IYCF policy. In 2014, the government was noted as the nodal actor in both policy development and implementation. The core of the nutrition network in 2014 was the Ministry of Health and Family Welfare (MoFHW), UNICEF, and the Bangladesh Breastfeeding Foundation (BBF), according to the 2014 Netmap analyses (see Rasheed et al., this issue). Interviewees perceived this change as important for both the acceptability and sustainability of nutrition programming. In general, respondents also perceived an increase not only in activity level but also in the number of actors focused on nutrition in Bangladesh since 2010. From the Net Map analysis, the government had the highest influence, followed by individual leaders and the media, and these three groups were also reflected in interviews, demonstrating their centrality in understandings of actor power within the policy community. Most actors mentioned were generally seen as positive players for the nutrition and IYCF agenda in Bangladesh, even though many still struggled with effective implementation, monitoring, and review of nutrition initiatives. A powerful group of other actors defined as negative influences for the nutrition agenda in Bangladesh were the formula companies. The perceived power of these companies was noted to have changed little, if at all, since 2010. Despite the leadership role assumed by the Bangladeshi government, individual leadership was reported to remain lacking; on multiple occasions, interviewees pointed to the need for individual leaders within government agencies and the medical profession to translate improved policy into action.

In Ethiopia, little seems to have changed over five years in terms of actors and their power in Ethiopia (NetMaps not shown). The Federal Ministry of Health (FMOH) remained the hub in terms of both centrality and influence among the actor networks, because of the mandate given by the council of ministers to lead and coordinate the national nutrition coordination body (NNCB); the Ethiopian Public Health Institute (EPHI) was the second most influential governmental body. There was a common perception of limited capacity for nutrition leadership in FMOH and insufficient drive to push forward nutrition and IYCF agendas. Civil society and donors were numerous and active in Ethiopia, trying to rally in support of the national program; these groups gained influence because of their involvement in the issues, but these actors were perceived as weak in formal influence (particularly with new legislation limiting NGO reach at the community level). Information sharing was mainly through working groups and forums that brought together the different key players, particularly nutrition technical working group (NTWG), a forum consisting of government partners, NGOs, UN agencies and donors, and the Maternal, Adolescent, Infant and Young Child Nutrition Working Group (MIYCNWG). These were both assigned high influence because the multi-agency fora is where technical issues on child nutrition were discussed, and decisions on policy issues and guidelines development are made owing to the wealth of knowledge coming from different partners.

In all three countries, the SUN networks – civil society, business, or others – were not mentioned in the 2014 actor networks, despite all three countries having engaged closely with the SUN movement. This could be because the Netmaps focused more on individual actors than on networks.

### Commitment through the policy cycle

In Vietnam, availability of international financial and technical resources for nutrition increased dramatically between 2010 and 2014, providing the momentum for important policy-level changes to occur in Vietnam; national funds were not as forthcoming, however, meaning that implementation at scale remains a challenge, especially looking forward. Enforcement of policy and legislation--new and old alike--was noted to still be lacking, posing a challenge for impact. Issues identified as barriers in 2010--including national-level leadership on nutrition and IYCF, cross-sectoral response, and national funding-- remained issues mentioned by interviewees in 2014, further hampering implementation of the new policies. The top layers of government paid enough attention to advocacy to change written policies and legislation, but lack of adequate government-allocated funding, or clear implementation and enforcement, suggests that these were not priorities further down the chain of command. Therefore, despite positive changes in policy and legal documents in Vietnam, action on stunting and breastfeeding was still seen to be internationally-led, and in particular, internationally financed, as government financial commitment for nutrition was cut by 65% in 2014.

The plethora of important governmental policy programs and initiatives instated in Bangladesh since 2010 reflected increased political attention to nutrition at the national level, concentrated in government programming. The NNS was seen to centralize and mainstream nutrition programming within the Bangladeshi government apparatus, and this demonstrated promise, but did not secure successful implementation or monitoring. Policy change was relatively recent, and it remains to be seen how well government leadership will be enacted through on-the-ground activities in Bangladesh, as challenges remained in rollout [24]. Finances and manpower capacity were two key resources perceived as presently insufficient to accomplish nutrition goals, as laid out in the deepened policy; most financial support for nutrition policy still was coming from international sources, which could undermine the perception of the government as the nodal nutrition policy leader.

In Ethiopia, government attention to nutrition was noted to be sporadic and limited to certain sectors close to the issues; nutrition has not become fully cross-sectoral or mainstreamed. While coordination was much-cited and the signing of the NNP by nine line ministries has been displayed as evidence of government commitment, subsequent work indicated that clarity on how coordination was to be achieved was still emerging [25, 29]. The government funding situation for nutrition--even through the health sector--was poor, and was non-existent in most other sectors, but it was hoped that the costed national nutrition plan under the NNP would help garner more resources. The budget allocation was described as minimal, with funding for nutrition coming mainly through international donors and co-operating partners, even while restricting regulations on donors and NGOs make direct program support difficult. Implementation of the NNP was starting to roll out in the decentralized system, and management, strategic and technical capacities in nutrition were noted challenges among human resources within the health system, along with low wages and high staff turnover. The FMOH was supported by technical assistance from INGOs and UN agencies who have seconded staff to the government, indicating that there was significant external, international funding and expertise being used to support nutrition efforts in Ethiopia.

### Contribution of Alive &Thrive

In Vietnam, stakeholders recognized A&T as a key player in a set of advocates for better nutrition and IYCF policies and programs. Over the four-year period, A&T undertook policy engagement through three broad strategies: engaging to set the agenda and discourse, popularizing the discourse through the media, and building capacity and engaging to build capacity. Activities within these included media outreach, evidence-based dialogue, gathering evidence, alliance building, capacity building, dissemination of evidence-based information for the purpose of capacity building or behavior change in support of a policy, and dissemination of research. A&T along with UNICEF was often cited as being among the most influential and active policy actors in support of changes to the policy environment for IYCF, and in particular breastfeeding. The process that led to revision of the maternity leave policy was previously documented, illustrating how change occurred [30]. It is likely that the strategic alliance made by A&T with UNICEF contributed to its fast rise to influence in the policy space in Vietnam. In general, those involved in IYCF policy advocacy in Vietnam recognized A&T’s role as pivotal, and A&T likely contributed to improvements in the policy context for IYCF in Vietnam.

In Bangladesh, A&T was recognized as being a vital actor both in advocacy and programming for IYCF over the past years. It was credited with having helped to orient society towards nutrition and IYCF as key issues, setting the perspective on nutrition that now dominates the policy sphere. Specific contributions noted included standardization of instruments, developing materials and active cooperation with, and alliance-building among, important actors working on IYCF. A&T was credited with successful media campaigns on IYCF, including networking with and the training of journalists, as well as providing technical information “in a non-technical way” to the population. In addition, A&T also provided technical support to the NNS in its early stages of implementation, developing the IYCF module and training master trainers. A&T was noted to have supported the government in taking a more nodal position on IYCF and nutrition policy, by recognizing the need for government centrality in nutrition programming to increase acceptance and sustainability. The sustainability of media programming, especially that of A&T, as well as limitations on journalists, however, remained challenges faced by the policy community in Bangladesh.

In Ethiopia, government, NGO and UN respondents recognized A&T as a key IYCF partner, raising IYCF as a major issue in national policies and among regional governments, and building on earlier efforts to address IYCF initiated by the AED/LINKAGES project between 2004 and 2006. A&T was most recognized for producing improved and simplified BCC materials on IYCF that have been approved by the FMOH and are widely used by health workers in facilities and health extension workers, as well as other development partners. Another important contribution of A&T was its influence on the NNP; A&T staff were noted to have played a key role in the revision of the IYCF sections within the NNP and working closely with other partners on the plan. Most government and NGO interviewees agreed that awareness of IYCF in the community increased because of an A&T-led media campaign at national and regional levels. A&T was also recognized for contributions to a shift of policy and programs to focus on stunting reduction; A&T was, however, perceived to have taken on too much and to have had limited opportunities for impact on actual implementation.

## Discussion

Despite varied contexts–political systems and actor networks, issue characteristics, and support for IYCF and nutrition--a set of focused advocacy activities to promote a set of nutrition actions based on technical evidence and international norms was able to support movement of national policy and legislation environments to be more conducive to improved IYCF and better nutrition for children. The findings show that much was achieved along this path, but that much remains to be done, especially to continue to support the implementation of policy and legislation, continued investments in supporting monitoring of supportive policies for breastfeeding, and creating system-wide commitment and capacity to implement programs.

The strategies used by A&T that were intended to promote key ideas and improve the policy and regulatory environment to create sustainable improvements in IYCF operated through at least four mechanisms that prior research has identified as being important for nutrition policy advancement. First, mobilizing a diversity of strategies, as A&T did, is important for strengthening commitment, coherence, consensus, and coordination in the advancement of nutrition agendas in countries; in particular, policy advancement requires planning and agenda formation as well as leadership and strategic capacity [7]. Second, strengthening political and system commitment requires sustained efforts from policy entrepreneurs and champions [8, 31], and was embodied in A&T’s alliance-building, policy dialogues, and media and dissemination strategies. Third, using and creating high-profile internal and external policy windows are important for bringing attention to nutrition [8]. Policy windows used or created by A&T and partners included award ceremonies, launch events, and special days or weeks. Fourth, external framing and advocacy of policy issues is an important factor for the formulation and sustainability of policy advancement [8, 19], and A&T engaged in efforts to popularize the discourse via media information dissemination in each country. Through these efforts in combination with other development projects, the written policy environment was changed to a great extent in each country.

There were differences as well as similarities between countries. In Vietnam, there have been some important changes relating to nutrition policy over the past six years, in particular ratification of the Maternity Leave Policy and the Advertising Law to restrict formula advertising. There are also dissenting voices, however, and remaining implementation and enforcement issues, particularly in the context of continued efforts to decentralize actions for nutrition [28]. In Bangladesh, mainstreaming through the NNS that has brought government into the nodal position for nutrition programming suggests increased possibilities to meet goals on nutrition and IYCF. Lack of resources and capacity, however, has inhibited the rollout of the full potential of nutrition policy and programming [22, 24], and enforcement particularly of the Code remains weak. In Ethiopia the new NNP is bringing coherence to a formerly disparate set of programs related to nutrition, and has started to move the country away from a set of policies and plans dominated by reaction to repeated emergencies, and to align its rhetoric with the international discourses on prevention and treatment of undernutrition. Ethiopia’s nutrition policy is not yet fully supported by sufficient budgets, operational plans, and implementation, findings that are also emphasized in other work [25]. In each country, therefore, we find increasing technical completeness of written policy and to the national nutrition discourse, which align with renewed global discourses of multi-sectoral action for stunting reduction, and improved exclusive breastfeeding along with limiting infant formula use. At the same time, in each country, practical and financial commitment that would allow these ideas to be enacted is still emerging.

Key actor groups identified in each country were government, civil society, development partners, and the private sector, with a mixed view of the role of the private sector in breastfeeding. In Vietnam, for instance, with its context of a rapid modernization and high participation of women in the work force, responses from many actors took a nuanced view citing the convenience of formula for working women. Interviewees from Vietnam and Bangladesh also cited multiple instances of interventions of the infant formula industry, from the advertising of breast milk substitutes directly, to the funding of government departments tasked with regulation. This issue is ongoing and is a key fault line between core nutrition policy actors and the private sector. Nutrition policy research is starting to engage with private sector involvement in nutrition through the food system [32]. Within the academic and civil society communities, however, there are differing views on the proper place for the private sector in IYCF; some think that it has no place and that its interests are too far out of line [33, 34], and others think that properly policed public-private partnerships are vital to filling resource gaps for nutrition and creating systems at scale [35]. Making the discourse more difficult are the lack of agreement on the meaning of terms such as “nutrition” and “public-private partnerships’ in discussions [32]. Overall, there has been a lack of evidence on the process and impact of private sector involvement in nutrition in general, and in IYCF in particular [2, 36, 37].

A key challenge for policy advocacy is the positioning of issues that are often not considered to be immediately pressing for political or electoral gains. Indeed, public policy researchers have previously highlighted differences between “pressing” and “chosen” problems in the policy process [38, 39]. Nutrition, particularly in its chronic form as stunting, is generally thought of as a chosen issue, in that it is not immediately pressing politically to address [1], and this challenge of positioning nutrition and IYCF emerged in several ways in our findings. Specifically, the issue of exclusive breastfeeding was commonly seen by respondents in this research as not politically urgent, with the international community generally leading advocacy to focus on these topics. This is similar to findings from a global landscape analysis of commitment to breastfeeding [40]. Our findings suggest that the key messages about the importance of addressing stunting and low breastfeeding rates, and some core actions to address these, are reaching the nutrition policy community (the internal frame). It is unclear in each case, however, how far this attention extends beyond the international actors and the more immediate circles of the national nutrition policy community, to the rest of government or to society at large (the external frame). In each country, the top layers of government were noted to have paid enough attention to the IYCF and nutrition advocacy case to change written policies and legislation. Ineach country, however, limited government-allocated funding, limited investments in implementation and enforcement plans, and constrained health-system delivery capacity for nutrition suggests that these are still emerging priorities throughout government. More efforts to highlight necessary investments to support implementation and legal enforcements are therefore likely to be necessary, though these needs were not the focus of the policy advocacy reported here. The issue of chosen versus pressing problems also raises the issue of legitimacy when global networks of civil society advocates are moving beyond traditional advocacy and towards participation in national policy and institutional change [41, 42], a key area for further study.

This nutrition policy-process research is novel in examining change in the context of ongoing strategic efforts to strengthen policy environments to support IYCF and nutrition in three different contexts. Other studies that have examined change in policy contexts have done so in places where active and well-resourced policy advocacy was not in place [43] and often in shorter windows rather than change over multiple years [44, 45]. Thus, our study provides insights into issues that groups advocating for nutrition–both global and national–will need to consider to strengthen the results of advocacy. The research used multiple methods to examine contributions of a policy advocacy intervention to national nutrition policy spheres. The evaluation of multifaceted interventions into complex systems is difficult, involving numerous actors and sub-systems, multiple aims, and shifting contexts; to address these, evaluations should assess context, components of programs, and connections between system actors and components, in addition to impacts [46], and our study has addressed each of these elements. We used a prospective design with an assessment of the policy environment at the beginning and end of the intervention period, as opposed to a retrospective analysis.

The five-year timespan of this research was long enough to track these components, and to start to see IYCF and stunting emerge onto the agenda and into written policy in each of these three countries, but was possibly too short to see change in more downstream stages of the policy cycle towards implementation. Policy and social change often occurs over decades, so follow-up will be needed to understand the process more fully. Although we show that networks in all three countries became more complex over time, it is difficult to ascertain using these methods whether there truly are more players entering the nutrition and IYCF space, or whether interviewees had just become more aware of existing actors due to increased advocacy activity. Finally, we note the obvious limitation of a lack of counterfactual to answer the question of “what would have happened in the absence of A&T”. In policy settings, this is close to impossible to address since randomizing advocacy interventions in a porous and well-connected policy field is infeasible. Through active tracking and triangulation of the types of activities undertaken by A&T to support the policy landscape, however, we were able to highlight some of the contributions of the initiative. Furthermore, in full recognition that policy and legislative change are affected by multiple factors, the goal of our study was predominantly to document changes and broad drivers over time, rather than to attempt to link specific advocacy activities to specific policy outcomes.

These changes and shifts in multiple factors affecting the ascendance of issues on policy agendas occurred in the context of a large and growing global movement for nutrition, with each of the countries being active participants in the Scaling Up Nutrition movement and with multiple global development partners playing active roles both in SUN and in the countries. An overall enabling environment for country shifts in priorities has been created by the emergence of a global narrative for nutrition and IYCF by SUN and associated entities (e.g. Thousand Days). Nevertheless, country stakeholders were able to articulate the strategic and practical roles played by A&T in supporting progress on one of the many intervention areas promoted by the SUN; this speaks, therefore, to the importance of nationally-focused and nationally-relevant advocacy engagement.

In conclusion, targeted policy-level interventions by external actors working in concert with in-country stakeholders and in an evidence-based, technically sound manner in support of national targets, can catalyze significant advancement of national policy environments. Our findings are in accordance with the small body of other work focusing on international nutrition advocacy [4, 30, 47] and public health advocacy more broadly [17], which has found that well-planned and well-implemented advocacy can bring significant change in aspects of national policy environments, even in short periods of time. The key issue going forward is whether the new and improved sets of policies and programs in each country will be fully enacted, as limited funding and enforcement present challenges for national governments. Further understanding of the aims and incentives of key actors, and in particular the interactions of private sector and government as a driver of IYCF policy enforcement, will be required to understand how best to intervene for strong implementation of policy in future. For the advocacy community, the lack of political urgency for nutrition and IYCF and questions over the legitimacy and role of international networks has implications for how far the issue of nutrition is likely to move in these countries and others.
